# Experimental Development of XR Enteral Feeding Function for an Endotracheal Suctioning Training Environment Simulator

**DOI:** 10.3390/s26051499

**Published:** 2026-02-27

**Authors:** Noriyo Colley, Shunsuke Komizunai, Atsuko Sato, Takanori Ishikawa, Mayumi Kouchiyama, Kazue Fujimoto, Toshiko Nasu, Ryosuke Nishima, Aiko Shiota, Eri Murata, Yumi Matsuda, Shinji Ninomiya

**Affiliations:** 1 Faculty of Health Science, Hokkaido University, Hokkaido 060-0808, Japan; 2 Faculty of Engineering and Design, Kagawa University, Kagawa 760-0016, Japan; komizunai.shunsuke@kagawa-u.ac.jp; 3 Department of Nursing, Faculty of Nursing, Hiroshima Bunka Gakuen University, Hiroshima 730-0037, Japan; a-sato@hbg.ac.jp (A.S.);; 4 Faculty of Medicine and Graduate School of Nursing, Yamagata University, Yamagata 990-0021, Japan; e.murata@med.id.yamagata-u.ac.jp (E.M.);; 5 School of Nursing, Faculty of Health Sciences, Hiroshima International University, Hiroshima 739-2631, Japan; s-nino@hirokoku-u.ac.jp

**Keywords:** XR, nursing education, technology-dependent children, simulation-based training, enteral nutrition

## Abstract

**Highlights:**

**What are the main findings?**

- The simulator uniquely integrates dosing-rate sensing devices with XR to present real-time patient reactions, including facial expression changes and cyanosis.
- Sensor-derived data enabled the quantitative measurement of procedural behaviors in both students and nurses.
- Projection mapping and tablet-based interfaces produced different perceptions of embodiment, visibility, and usability among learners.

**What are the implications of the main findings?**

- The system provides a foundation for assessing aspects of nursing skills that have traditionally been considered tacit or difficult to quantify.
- Objective, sensor-based performance metrics may support more standardized and data-driven approaches to enteral-feeding education.
- Insights from interface comparisons can guide future XR design choices to optimize learner engagement and instructional effectiveness.

**Abstract:**

Background: Existing XR simulators for enteral feeding rely mainly on self-reported learning outcomes and procedural checklists. As a result, they offer limited opportunities to capture objective behavioral data or to present dynamic patient reactions. This two-stage pilot study evaluated an XR-based gastrostomy tube-feeding simulator (ESTE-TF) that integrates sensor-derived performance metrics and two biological-reaction presentation modalities (projection mapping and tablet display). Methods: In Experiment 1, nursing students completed pre- and post-experience questionnaires assessing perceived learning across seven domains, alongside sensor-based measurements of feeding-start timing, dosing-rate characteristics, and total procedure time. Experiment 2 employed a tablet-based version with four learning items assessed for students and post-experience evaluations collected from registered nurses. Participants also compared the two XR presentation methods. Results: Students demonstrated perceived learning gains of small-to-large magnitude across both experiments (Experiment 1: d = 0.455–0.974; Experiment 2: d = 0.014–0.886), with wide 95% confidence intervals reflecting the exploratory nature of this pilot work. Sensor-derived data showed greater dosing-rate variability and longer procedure times among students than nurses. Participants reported that projection mapping offered a more embodied experience, whereas tablet displays provided clearer visibility. Conclusions: These findings indicate the feasibility and preliminary educational potential of integrating sensing technologies with XR-based biological-reaction presentation for gastrostomy-feeding training. Given the small samples and non-validated measures, results should be interpreted as exploratory. Future research will refine sensor accuracy, establish standardized performance metrics, and evaluate learning outcomes using validated instruments and controlled study designs.

## 1. Introduction

**Figure 1:**
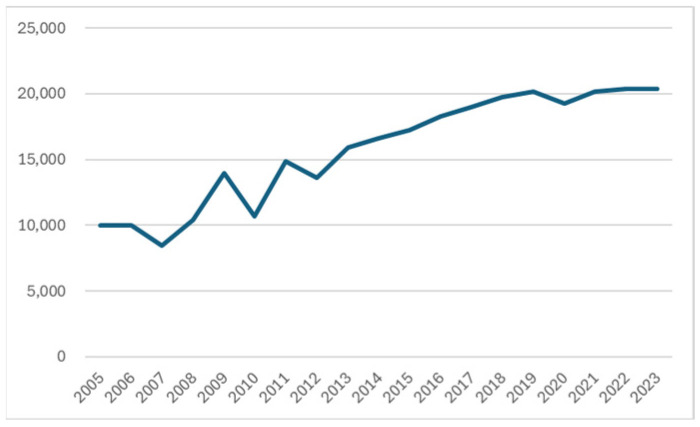
Estimation of the number of technology-dependent children (MHLW).

Japan is currently facing an urgent shortage of home care services, driven by the growing number of technology-dependent children. According to the Act for Children Who Require Healthcare Support and Their Family Support, enacted in 2021, a technology-dependent child is defined as a child who requires constant healthcare support in order to carry out daily and social activities. This includes children under 18 years of age and those 18 years of age or older who are enrolled in high schools, the upper division of secondary schools, and the upper division of special needs schools, as defined by the School Education Act [1]. The Ministry of Health, Labour and Welfare (MHLW) reported in the Status of Technology-Dependent Child Support Centers that the number of technology-dependent children in Japan is estimated to have doubled over the 15 years from 2005 to 2020, despite the low birth rate [2] (Figure 1).

The Ministry of Internal Affairs and Communications (MIC) has pointed out that support measures are essential, considering the opinions of prefectural boards of education that it is difficult to secure nurses due to low salaries, concerns about the working environment in non-healthcare settings, and an unclear role at special needs schools [3].

The most common reason for needing healthcare services for these technology-dependent children was “congenital illness” at 63.8%, followed by “acquired illness” (19.7%), “accident” (5.3%), and “other” (11.2%); “other” responses included “problems during birth,” “very low birth weight,” “unknown cause,” and “under investigation” [4]. Some low-birth-weight babies admitted to the NICU require continued care after discharge. Looking at the breakdown of healthcare required, 74.4% needed tube feeding (nasogastric, gastrostomy, and enterostomy), 69.0% needed suctioning (tracheal, oral, and nasal), and 33.0% needed home mechanical ventilation [4]. This trend is observed not only in Japan. Berry et al. (2011) noted that neurological impairment (57%) and gastrostomy tubes (56%) were the main reasons for medically dependent children [5]. Aitchison et al. (2020) pointed out that family problems, fragmentation of health and care provision, psychological difficulties, or social issues could be factors for poor outcomes in children with complex health needs (CHNs) [6].

Advances in percutaneous endoscopic gastrostomy and the growing shift toward cost-efficient, community-based models of care have expanded the number of patients eligible for long-term home enteral nutrition (HEN) [7]. Although epidemiological evidence remains limited, recent reports indicate a notable rise in the number of children receiving HEN. For example, national data from Poland documented an increase from 104.1 per million children in 2010 to 270.3 per million in 2018 [8].

As HEN becomes more common, the need for safe and effective gastrostomy-feeding education continues to grow. At the same time, the ongoing shortage of nursing staff has reduced opportunities for supervised on-the-job training (OJT) in many clinical settings. Consequently, novice nurses and students have fewer chances to observe and practice gastrostomy tube feeding under expert supervision. This situation highlights the need for scalable, standardized simulation-based training tools that can supplement diminished clinical learning opportunities. However, existing enteral-feeding simulators primarily focus on procedural accuracy and lack haptic learning components [9].

Extended reality (XR) technologies—such as virtual, augmented, and mixed reality—have been increasingly applied in healthcare education, yet current systems face limitations. VR-based prototypes do not integrate with physical manikins, AR systems struggle with alignment and limited fields of view, and MR approaches have rarely incorporated realistic patient reactions. However, dynamic patient responses—such as facial expressions or cyanosis—are still largely absent across these modalities. These reactions are essential for detecting complications, including coughing or respiratory distress. Moreover, current XR and high-fidelity simulators rely predominantly on self-reported learning outcomes and procedural checklists, and they generally lack mechanisms to capture objective behavioral data or to present dynamic physiological reactions during the procedure. As a result, existing systems provide limited insight into learners’ real-time decision-making processes—such as dosing-rate control, recognition of patient discomfort, or timely adjustment of procedures—which are essential for safe enteral-feeding practice.

To address these gaps, the present two-stage pilot study evaluates an XR gastrostomy tube-feeding simulator developed through cross-disciplinary collaboration. The simulator integrates sensor-based skill measurement, dynamic biological-reaction mapping, and two presentation modalities (projection mapping vs. tablet display). Therefore, this study aimed (1) to evaluate the usability and perceived educational value of the simulator and (2) to examine how the two presentation methods influence learner cognition and engagement.

## 2. Materials and Methods

### 2.1. Materials

**Figure 2:**
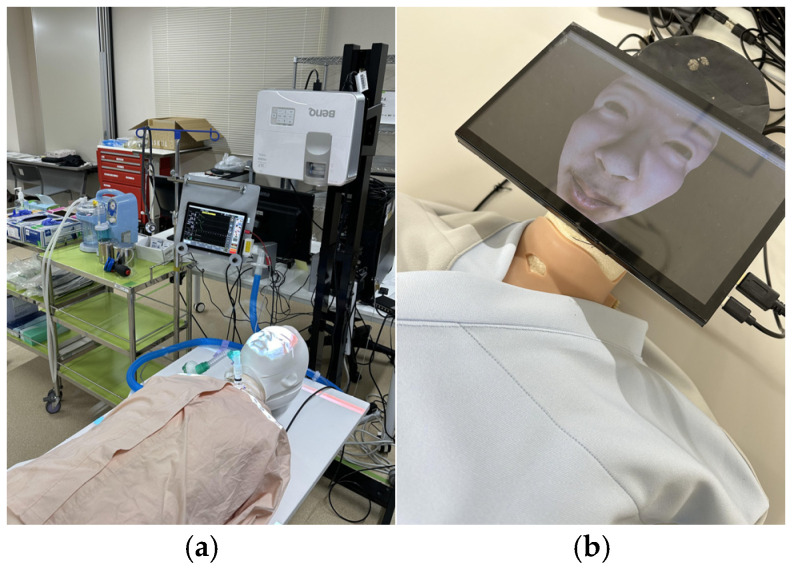
Comparison of ESTE-TF using two biological response presentation methods: (**a**) projection mapping, and (**b**) ESTE-TF using a tablet.

**Figure 3:**
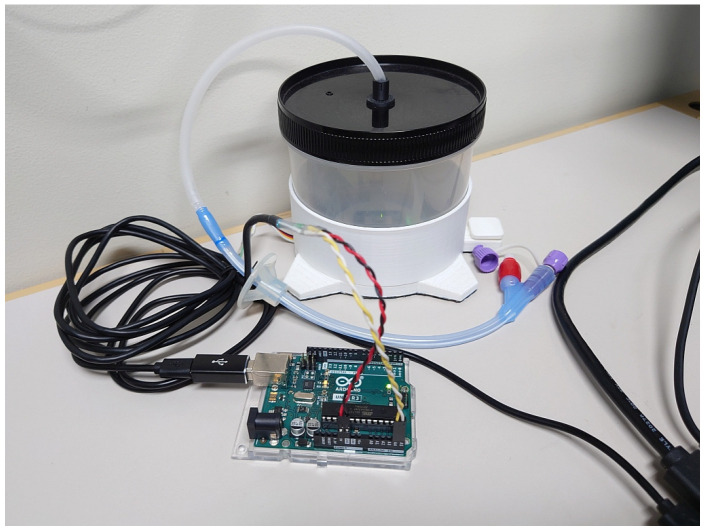
A sensing device used to measure the dosing rate in ESTE-TF.

The device used in this research is named ESTE-TF (Endotracheal Suctioning Training Environment SIMulator: Tube-Feeding function added version) (Figure 2 and Figure 3) [10,11]. ESTE-SIM comprises four components: a sensor that measures nursing techniques, a microcomputer (Arduino Uno R3, Arduino Holding, Monza, Italy) that acquires sensor data, a computer that generates the patient’s facial expressions and biological reactions based on the sensor data, and a projector (MW632ST, BenQ, Taipei, Taiwan) or a tablet that displays the patient’s facial expression. A weight sensor (M5Stack Unit Mini Scales, M5Stack Technology Co., Ltd., Shenzhen, China) and a drain tank (Daiso Industries Co., Ltd., Hiroshima, Japan) are added for the tube-feeding function. Standard gastrostomy tubes, infusion tubes, gastrostomy bottles, and stands can be used for tube-feeding training. By connecting the gastrostomy tube to the drain tank and measuring the weight of the drain tank (the amount of weight changes), it is possible to quickly determine whether the tube-feeding dosing rate is appropriate. For example, if the dosing rate is faster than a set value, feedback can be provided, such as by changing the patient’s facial expression to look distressed. Because the simulator is still in the development stage, the specific numerical thresholds and calibration parameters used to trigger patient-response animations are not yet finalized; however, the system relies on predefined sensor-based deviations from safe feeding parameters, refined through iterative expert review to ensure face validity.

### 2.2. Participants and Questionnaires

Nursing university students and registered nurses experienced the enteral-feeding simulator (ESTE-TF, Figure 2 and Figure 3) and completed a questionnaire. After a brief orientation on gastrostomy tube feeding, participants performed the procedure using the simulator. Because no standardized scale exists for XR-based enteral-feeding simulators, we developed original questionnaire items aligned with key domains in competency-based nursing education: self-efficacy, clinical judgment, and communication competence. Nursing students completed both pre- and post-experience questionnaires, whereas registered nurses, who already had clinical experience, provided only post-experience usability evaluations.

In Experiment 1, the pre-experience questionnaire consisted of seven items addressing (1) observation of key points, (2) confidence in performing the procedure, (3) identification of important care points, (4) risk prediction, (5) troubleshooting ability, (6) assessment of the gastrostomy insertion site, and (7) communication with the patient. The same items were administered after the experience to assess changes in perceptions. In addition, the post-experience questionnaire included one item asking, “Do you think that changes in facial expression enable ethical practices such as speaking in a way that respects the patient’s dignity?”

In Experiment 2, four simplified items—(2) confidence in performing the procedure, (5) troubleshooting ability, (6) assessment of the gastrostomy insertion site, and (7) communication with the patient—were used to minimize respondent burden. The post-experience questionnaire additionally included two items comparing the two biological-reaction presentation methods (projection mapping vs. tablet) in terms of ease of viewing and perceived impersonality.

All items in both experiments were rated on a 4-point Likert scale (1 = strongly disagree, 4 = strongly agree). Internal consistency was examined using Cronbach’s α. Free-text responses were analyzed using simple content analysis to identify common themes. For statistical comparison, an F-test was conducted to confirm equal variance, followed by paired *t*-tests.

### 2.3. Recruitment

**Figure 4:**
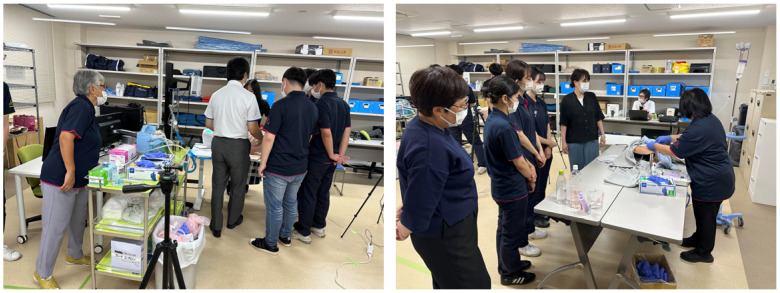
Overview of SIMweek 2025 with Simmar+ESTE-SIM (**left**) and ESTE-TF (**right**). Annual multi-institutional simulation exercises using Simmer+ESTE-SIM (ver. 1.0.3) have been conducted as part of a collaborative research project across several universities; 2025 marked the third iteration.

Participants were recruited through the events held during SIMweek 2024 and 2025 (Figure 4).

SIMweek refers to annual simulation-education events held at the Clinical Training Center of Chugoku Rosai Hospital in Hiroshima, Japan. These events were initiated in 2023 under the leadership of the hospital director, Dr. Kaoru Kurisu, as part of an interdisciplinary, multi-institutional collaboration aimed at developing and evaluating innovative simulation-based educational tools. In recent years, the initiative has also attracted interest from Hiroshima Prefecture, and the activities have gradually expanded into a broader industry–academia–government partnership. Participants were recruited from nursing programs, hospitals, or school settings, with collaborating university faculty assisting in recruitment. Although participation has expanded to include clinical engineers and nurse practitioners, the present study analyzed data only from nurses and nursing students. To minimize potential evaluator bias, the performance checklist was administered anonymously via Google Forms, ensuring that instructors involved in recruitment could not identify individual respondents.

## 3. Results

Research participants included nursing students from a university and experienced nurses who had more than three years of experience at the time of SIMweek 2024 and 2025.

### 3.1. Result

#### 3.1.1. Experiment 1: SIMweek 2024

1. Participants

Participants were recruited according to predefined eligibility criteria. The nursing student group consisted of students enrolled in an accredited fourth-year undergraduate program with a specific emergency nursing course, who were able to provide informed consent. The registered nurse group included licensed nurses currently employed in clinical practice with at least one year of professional experience and the capacity to provide informed consent. Individuals who did not agree to participate in the simulation experiential session were excluded from the study. The actual number of participants is shown in Table 1.

2. Data Analysis of Learning Assessment

**Figure 5:**
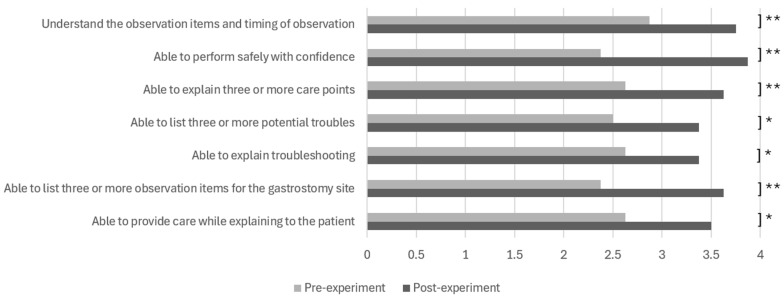
Nursing students’ learning outcomes (paired *t*-test, *: *p* < 0.05, **: *p* < 0.01) in SIMweek 2024.

**Figure 6:**
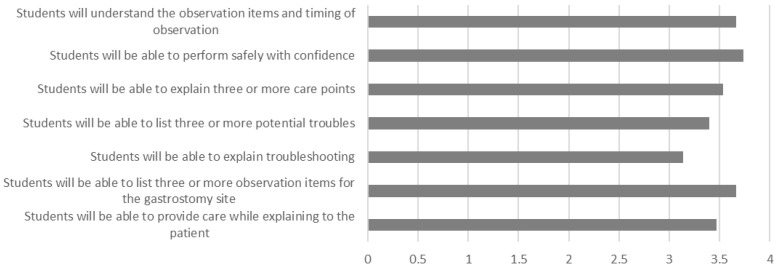
The mean of nurses’ perception of the simulator after experience.

The projection mapping version of ESTE-TF (Figure 2a) was used in this study. For the nursing student group, a questionnaire was administered before and after the simulator experience to assess changes in learning outcomes (Figure 5). For the nurse group, a post-experience questionnaire was administered to evaluate nurses’ perceptions of the simulator after the experience (Figure 6). The open-ended responses were analyzed by categorizing the comments according to thematic content.

There were statistically significant differences in all items between the student group before and after the experience, including observation during tube feeding (*p* = 0.003, d = 0.603, 95% CI [−0.090, 1.296]), gaining confidence (*p* = 0.003, d = 0.974, 95% CI [0.281, 1.667]), understanding important points (*p* = 0.009, d = 0.653, 95% CI [−0.040, 1.346]), risk prediction (*p* = 0.010, d = 0.529, 95% CI [−0.164, 1.222]), troubleshooting methods (*p* = 0.040, d = 0.455, 95% CI [−0.238, 1.148]), observation of the gastrostomy site (*p* = 0.001, d = 0.817, 95% CI [0.124, 1.510]), and explaining to the patient (*p* = 0.021, d = 0.569, 95% CI [−0.124, 1.262]). Cronbach’s alpha for the scale was 0.834, indicating good internal consistency despite the small sample size.

The results of the post-experience survey of the nurses showed that the learning benefits of the ESTE-TF were highly rated, with an average score of 3 or above in all areas, including observation during tube feeding (mean = 3.667), gaining confidence (3.733), understanding important points (3.533), risk prediction (3.4), troubleshooting methods (3.133), observation of the gastrostomy insertion site (3.667), and explanations to patients (3.467).

In response to the survey question, “Do you think that changes in facial expression enable ethical practices such as speaking in a way that respects the patient’s dignity?” eight nurses (50%) answered “strongly agree” and eight nurses (50%) answered “agree.” No one responded “disagree” or “strongly disagree.”

#### 3.1.2. Experiment 2: SIMweek 2025

1. Participants

As for the inclusion/exclusion criteria, the nursing student group comprised individuals enrolled in an accredited undergraduate nursing program who had completed at least one clinical practicum and provided informed consent. The registered nurse group consisted of licensed nurses with a minimum of one year of clinical experience who were able to provide informed consent. Individuals who declined participation in the simulation-based experiential session were excluded. The second- and fourth-year student groups were combined into a single student group. The actual number of participants is shown in Table 2.

2. Data Analysis of Learning Assessment

The tablet version of ESTE-TF (Figure 2b) was used in this study. To simplify the evaluation criteria, the number of questions in the survey was reduced from seven in the previous year to four. A questionnaire for nurses was administered after the experience, and after calculating basic statistics, the free comments were categorized by content.

**Figure 7:**
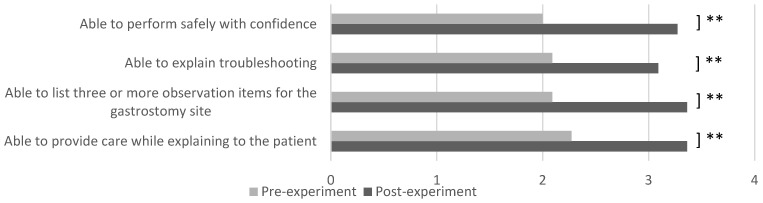
Nursing students’ learning outcomes (paired *t*-test, **: *p* < 0.01) in SIMweek 2025.

Survey results indicated that the student group showed significant improvements in all learning areas when comparing pre- and post-experience scores (Figure 7), including confidence acquisition (*p* = 0.003, d = 0.739, 95% CI [0.148, 1.330]), troubleshooting strategies (*p* = 0.001, d = 0.014, 95% CI [−0.577, 0.605]), observation of the gastrostomy site (*p* = 0.000, d = 0.662, 95% CI [0.071, 1.253]), and the ability to explain procedures to patients (*p* = 0.000, d = 0.886, 95% CI [0.295, 1.477]). Although the sample size was small, the effect sizes and their wide confidence intervals reflect the exploratory nature of this pilot study and indicate varying degrees of perceived learning gains. The scale demonstrated good internal consistency, with a Cronbach’s alpha of 0.833.

In a survey about the ease of viewing projection mapping and tablets, the results showed that tablets looked more impersonal than projection mapping (mean = 3), and that it was easier to see the facial expression changes on tablets than on projection mapping (mean = 3.125). This indicates that tablets looked impersonal, but the images were easier to view.

Open-ended responses were predominantly positive. One student noted that the simulators for tube feeding and ventilator suctioning were easy to use because changes in facial expressions were clearly observable, allowing for deeper learning of procedures not experienced during clinical practice (S10, second-year student). Another reported that performing the simulation themselves enhanced their understanding of procedural steps and points requiring attention (S4, fourth-year student). A second-year student valued the opportunity to visually observe and experience scenarios resembling real emergency care, an area of personal interest (S8, second-year student). Others emphasized that, because students cannot provide direct patient care, the simulation helped them better visualize clinical situations (S11, fourth-year student).

Post-experience survey results from the nurse group included the following comments: “It would be great if there was a feature where patient AI could answer students’ questions” (N3); “I think it can also be applied to nurses with a gap in their work experience, such as the nursing association’s seminars for gap-year nurses” (N4); “I like that it can be combined with a suction function, and I think adding the ability to lift the bed when using tube feedings would make for a more realistic experience” (N6); “If nursing students could instantly know the amount of dosing, they could make corrections immediately” (N9); “There was a part of the tube where negative pressure was applied, making it difficult to inject; it might be better to restrict the flow using a check valve or clip” (N10); “It’s revolutionary to be able to measure things like weight, but since nurse educators can observe the dosing speed, it may not be necessary” (N11); and “Nursing students can actually administer tube feedings. It would be nice if they could also auscultate the bowel sounds from the simulator” (N12).

**Figure 8:**
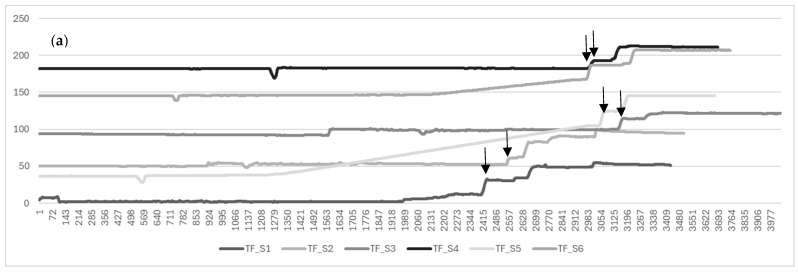

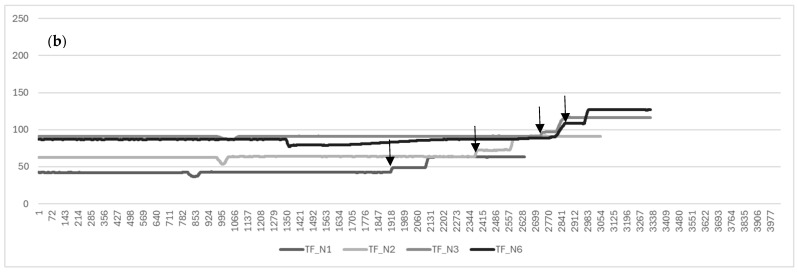
Comparison of proficiency between the nursing student group (**a**) and the registered nurse group (**b**). Downward arrows indicate the sensor-detected onset of dosing. The differing initial weights reflect residual values from previous measurements, suggesting the need for a future zero-point calibration feature.

Next, we attempted to evaluate learning using the ESTE-TF sensors. The vertical axis of Figure 8 represents the amount of tube feeding administered, and the horizontal axis represents the timeline. The upper graph shows the data for the student group (a), and the lower graph shows the data for the nurse group (b). After excluding data for which sensing was not performed properly, there were six students in the student group and four in the nurse group. Comparing the sensing data for the student group and the nurse group, the total time required for the student group was statistically significantly longer in the student group (*p* = 0.004). Figure 8 shows individual line graphs for each participant, plotting weight over time and using changes in weight to represent drip rate. 

The downward arrow in Figure 8 indicates the time when the tube feeding started. In some cases, it can be clearly determined, but in cases like S5, where the gradient is gradual, it is difficult to determine. It is highly likely that the administration continued due to the loosening of the clamp that controls the speed, and further investigation into the cause is required.

## 4. Discussion

This pilot study explored the feasibility and preliminary educational potential of the ESTE-TF simulator, which integrates extended reality and sensing technologies for gastrostomy tube-feeding training. Across both experiments, students reported perceived improvements in multiple learning domains, including observation of key points, confidence in performing the procedure, identification of care priorities, risk prediction, troubleshooting, assessment of the gastrostomy site, and patient communication. Effect sizes in Experiment 1 ranged from moderate to large (d = 0.455–0.974), whereas Experiment 2 showed substantial variability (d = 0.014–0.886), with wide confidence intervals in both cases. These patterns are consistent with early-stage pilot studies with limited statistical power and indicate preliminary but consistent perceived learning gains. The learning assessment scale demonstrated high internal consistency in both years, supporting the reliability of the questionnaire used in this developmental phase.

Sensor-derived measurements further distinguished procedural patterns between nursing university students and expert nurses, particularly in total procedure time, suggesting that the system can quantify meaningful differences in performance. The comparison of biological-reaction presentation methods indicated that tablet-based displays were perceived as clearer but more impersonal, whereas projection mapping offered a more embodied and immersive experience. These findings highlight the importance of balancing realism, usability, cost-effectiveness, and cognitive load when designing XR-based educational tools.

The broader literature on XR-based simulation supports the potential educational value observed in this study. Prior research has shown that XR environments can enhance procedural accuracy, situational awareness, and learner confidence through repeated practice in a safe and controlled setting [12,13]. The positive responses from both university students and expert nurses in the present study align with these findings and suggest that dynamic biological reactions—such as facial expression changes [14,15] and cyanosis—may support patient-centered communication and clinical judgment, even though these constructs were not directly measured with validated instruments.

The incorporation of sensing technologies addresses a longstanding challenge in healthcare education: the difficulty of evaluating tacit psychomotor skills objectively. Previous studies have emphasized the need for data-driven assessment tools [16,17,18]. The present findings extend this work by demonstrating that ESTE-TF can capture measurable differences between novice and experienced practitioners. Future development should explore more comprehensive sensor-based indicators and integrate video-based activity recognition to enhance the robustness of performance assessment [19]. Enteral nutrition flow-rate sensors function as key reference metrics for thresholding biological-reaction display and quantifying procedural competence.

Participant feedback also identified several areas for refinement, including the real-time display of dosing speed and infused volume, improved flow-control mechanisms, and additional physiological cues such as bowel-sound auscultation. These responses further suggest the breadth of assessment parameters nurses draw upon when evaluating a patient’s condition. Accordingly, additional investigation is warranted to clarify the specific clinical judgment criteria nurses employ during enteral nutrition administration. As no prior studies have reported the use of sensing data in tube-feeding simulators, these insights will guide iterative improvements to the system.

Our developed nursing XR simulator clearly depicted the differences between the two groups depending on the proficiency of tube-feeding skills. However, this study has several limitations. It was conducted at a single site using convenience sampling, and the evaluation was short-term with a small number of participants. The student cohort included mixed academic years, which may have introduced variability in baseline preparedness. Technical issues with sensing accuracy also indicate the need for further refinement. Moreover, safe enteral-feeding practice requires multiple competencies—including observation, clinical judgment, and communication—that cannot be fully captured through dosing-rate characteristics alone. Future research should therefore focus on improving sensing precision, developing multidimensional performance metrics, and conducting larger multi-institutional and longitudinal studies to examine skill retention and transferability to clinical practice.

## 5. Conclusions

This pilot study examined the feasibility of the ESTE-TF simulator, which integrates extended reality and sensing technologies for gastrostomy tube-feeding education. Nursing university students demonstrated perceived improvements across multiple learning domains, and the assessment scale showed high internal consistency. Expert nurses also valued the visualization of dynamic biological reactions—particularly facial expression changes—as a means of supporting patient-centered communication and accurate clinical judgment. Sensor-derived measurements further distinguished procedural patterns between nursing university students and expert nurses, suggesting the potential of the system to capture aspects of tacit clinical skills that are difficult to evaluate using traditional methods like OJT. Future development should enhance physiological realism and usability, including real-time display of the dosing rate and infused volume, as well as additional functions such as bowel-sound auscultation.

## Figures and Tables

**Table 1:** Recruitment of participants in SIMweek 2024.

SIMweek 2024	Number of Participants/ Recruitment	Participation Rate
Fourth-year nursing students S1–S8	8/9	88.89%
Registered nurses N1–N16	16/16	100%

**Table 2:** Recruitment of participants in SIMweek 2025.

SIMweek 2025	Number of Participants/ Recruitment	Participation Rate
Second-year nursing students S6–S10	5/6	83.33%
Fourth-year nursing students S1–S5, S11	6/6	100%
Registered nurses N1–N11	11/12	91.67%

## Data Availability

The dataset is not yet publicly available, as it contains data whose analysis has not yet been completed.

## Abbreviations

The following abbreviations are used in this manuscript:

ESTE-TF	Endotracheal Suctioning Training Environment Simulator—Tube Feeding
TF	Tube Feeding
HEN	Home Enteral Nutrition
XR	Extended Reality, Cross Reality
